# Clinical Application of CT Navigation in treatment of Lumbar Spondylolisthesis with Minimally Invasive Surgery - Transforaminal Lumbar Interbody Fusion

**DOI:** 10.12669/pjms.36.5.2341

**Published:** 2020

**Authors:** Ru-de Sui, Chun-guo Wang, Jin-cai Zhang, Hai-tao Wang

**Affiliations:** 1 Ru-de Sui, Department of Imaging, Linyi Central Hospital, Linyi, 276000, Shandong, P. R. China; 2 Chun-guo Wang, CT/MRI Room, Women’s and Children’s Health Care Hospital of Linyi, 276000, Shandong, P. R. China; 3 Jin-cai Zhang, Department of Imaging, Linyi Central Hospital, Linyi, 276000, Shandong, P. R. China; 4 Hai-tao Wang, Department of Imaging, Linyi Central Hospital, Linyi, 276000, Shandong, P. R. China

**Keywords:** MIS-TLIF, Intraoperative CT, Lumbar spondylolisthesis

## Abstract

**Objective:**

To explore the clinical effect of the application of CT navigation in the treatment of lumbar spondylolisthesis with minimally invasive surgery - transforaminal lumbar interbody fusion (MIS-TLIF).

**Methods:**

A retrospective study was conducted on 30 patients with lumbar spondylolisthesis who were continuously treated in linyi central hospital from May 2018 to March 2019.The patients were divided into two groups,15 patients treated with MIS-TLIF with the aid of CT navigation during the operation were included into an observation group. Another 15 patients were treated with open transforaminal lumbar interbody fusion as the control group. The baseline information, including gender, age and course of disease, perioperative period and imaging conditions, and VAS and ODI scores of patients in the two groups were collected and analyzed.

**Results:**

Fifteen patients were included into the observation group, including 9 male and 6 female patients with an average age of 52.60 ± 6.31 and a course of disease of 16.33 ± 6.00 months. The other 15 patients were included into the observation group, including seven male and eight female patients with an average age of 52.87 ± 7.38 and a course of disease of 19.13 ± 9.89 months. The difference in the gender, age and course of disease between the two groups had no statistical significance (*P* > 0.05). However, the difference in the duration of operation and intraoperative blood loss between the two groups had statistical significance (*P*< 0.05). There were no statistically significant differences in wound complications, neurological complications, preoperative slippage rate, postoperative slippage rate, slippage reduction rate and screw placement accuracy (*P* > 0.05). VAS scores of the two groups were statistically significant from six months after surgery (P < 0.01). There was no significant difference in ODI between the two groups at any time point (P >, 0.05). VAS and ODI scores were improved at each time point compared with those before surgery.

**Conclusion:**

The minimally invasive transforaminal lumbar fusion performed with the aid of CT navigation during the operation shortens the duration of operation and the amount of bleeding, reduces the back pain, is beneficial to the early postoperative functional exercise, and speeds up the postoperative recovery.

## INTRODUCTION

Lumbar spondylolisthesis is a common spinal disease usually defined as the relativistic displacement between two adjacent cones of the lumbar spine, which breaks the order of the vertebral body sequence. Recurrent back pain and/or neurological symptoms are often caused by the disruption of the lumbar sequence. Transforaminal interbody fusion (TLIF) and posterior interbody fusion (PLIF) are common fusion surgical methods for the treatment of lumbar disease. However, the traditional TLIF surgical method has greater stimulation to muscle and soft tissue stretching. In recent years, with the development of Minimally invasive surgery technology, Minimally Invasive Surgery - Transforaminal Lumbar Interbody Fusion (MIS-TLIF) is becoming more and more widely clinical applications. In many studies, MIS-TLIF surgery has obvious advantages over open TLIF surgery, such as better retention of the posterior spinal structure, less intraoperative bleeding, postoperative pain, etc.^1^-^3^ Compared with the traditional posterior lumbar interbody fusion (PLIF), MIS - TLIF has the advantages of short operation time and small intraoperative nerve stimulation,^4^-^6^ but it has the disadvantages of small operation field and difficult operation, so it needs multiple fluoroscopy during operation, which prolongs the operation time. In this study, CT navigation technology was used in MIS-TLIF operation to explore the clinical application effect compared with traditional TLIF operation.

## METHODS

Thirty patients with lumbar spondylolisthesis treated in Linyi Central Hospital from May 2018 to March 2019 were selected. Fifteen patients treated with MIS-TLIF with the aid of CT navigation during the operation were included into an observation group. The other 15 patients treated with open transforaminal lumbar interbody fusion were included into a control group.

### Ethical Approval

The study was approved by the Institutional Ethics Committee (February 6, 2020) of Linyi Central Hospital, and written informed consent was obtained from all participants.

### Inclusion Criteria

(1) patients clinically diagnosed with lumbar spondylolisthesis; (2) patients with a slippage degree of I~II degree according to Meyerding typing as indicated by imaging evidence;^7^ (3) patients with single segment slippage; (4) patients in whom conservative treatment was ineffective for at least 3 months; and (5) patients with clinical signs consistent with imaging.

### Exclusion Criteria

(1) patients complicated with tumor, tuberculosis and infection or with congenital malformation; (2) patients with a previous history of lumbar surgery; (3) patients with a history of lumbar injury; (4) The patient had contraindications to the operation.

This study met the requirements of the *Declaration of Helsinki*; all patients signed the operation agreement.

### Operation Methods Observation group (MIS-TLIF)

(1) Surgery performed under general anesthesia, the patient was placed in the prone position; the operation area was sterilized; and a surgical drape was spread. (2) The reference frame is mounted on the posterior superior iliac spine of the patient, and the reflecting ball is within the receiving range of the binocular infrared camera. A three-dimensional (3D) CT scan of the patient’s lumbar spine was performed; and the results of image scan were entered into image navigation workstation. (3) A 2.0-4.0 cm longitudinal incision was made by connecting the surface projection points of the upper and lower pedicle of the operative target intervertebral space; (4) Use expandable casing to expand step by step and insert the working channel, install the fixed free arm and cold light source. (5) Spinal decompression surgery and discectomy were performed; Cage was inserted into the intervertebral space; bilateral screws were placed; the prebent hold-down bars were installed; the slippage centrum was lifted and restored; good reduction was confirmed. (6) A drainage tube was placed and the wound sutured.

### Control group (TLIF)

(1) Surgery performed under general anesthesia; the patient was placed in the prone position; the operation area was sterilized; and a surgical drape was spread. (2) Posterior midline incision was made; the skin and subcutaneous tissue were incised layer by layer to expose the supraspinous ligament. The paravertebral muscle was incised along the spinous process; and blunt dissection was performed. (3) After the insertion of pedicle screw with the aid of C-arm fluoroscopy, the position was confirmed to be satisfactory. (4) Spinal canal decompression was performed by lateral articular process approach; interbody fusion cage was inserted; after the internal fixation and fusion cage were confirmed by fluoroscopy to be in good position, the slippage vertebra was pulled and reduced. (5) A drainage tube was placed and the wound was sutured.

The operation on patients in the two groups was performed by the same group of physicians. The patients received conventional anti-inflammatory and dehydration treatment after the operation.

### Statistical Indicators

The Oswestry disability index (ODI), visual analogue scores (VAS) of the waist before the operation and one week, one month, three months, six months and one year after the operation in the two groups were counted.

### Imaging observation indicators

(1) Andrew staging^8^ was used to evaluate the accuracy of pedicle screw insertion (i.e. the screw completely in the pedicle cortex: excellent, ≤ 2 mm out of the cortex: good, 2~4 mm: medium, and > 4 mm: poor). (2) Preoperative and postoperative slippage rate in the two groups, reduction rate = (postoperative slippage rate - preoperative slippage rate)/postoperative slippage rate×100%.^9^

Perioperative complications include bleeding, with post operative complications such as poor wound healing and neurological complications. Neurological complications are aggravated original nerve symptoms such as pain, numbness and weakness after nerve root or dura injury.

### Statistical Methods

The software SPSS 21.0 was used for statistical analysis. Shapiro-Wilk method was used for normality test. The normally distributed measurement data were expressed as (χ±s), independent sample t test was used for comparison between the two groups; the variance analysis of repeated measurements was used for the comparison of measurement data at different time points. x^2^ test or Fisher exact probability method was used for the comparison of classified data. The difference was statistically significant for P < 0.05.

## RESULTS

Fifteen patients were included into the observation group, including nine male and six female patients with an average age of 52.60 ± 6.31 and a course of disease of 16.33 ± 6.00 months. The other 15 patients were included into the observation group, including 7 male and 8 female patients with an average age of 52.87 ± 7.38 and a course of disease of 19.13 ± 9.89 months. The difference in the gender, age and course of disease between the two groups had no statistical significance (P > 0.05). Table-I.

**Table-I:** General Data of Patients in the Two Groups.

	Gender		Age (years)	Course of disease (month)

	Male	Female		
Observation group (n = 15)	9	6	52.60±6.31	16.33±6.00
Control group (n = 15)	7	8	52.87±7.38	19.13±9.89
Statistical value		t=-0.106	-0.937	
P value	0.715^a^	>0.05	>0.05	

***Note:***

a is Fisher exact probability method.

The duration of operation and intraoperative blood loss in the observation group were 107.26±10.46 min and 222.33±46.87 ml, respectively; the duration of operation and intraoperative blood loss in the control group were 122.20±21.67 min and 459.87±80.41ml, respectively. The difference in the duration of operation and intraoperative blood loss between the two groups had statistical significance (*P* < 0.05). There was no statistical difference in wound complications, nerve complications, preoperative slippage rate, postoperative slippage rate, reduction rate and screwing accuracy (*P* > 0.05). Table-II.

**Table-II:** Perioperative Conditions of Patients in the Two Groups.

	Duration of operation (min)	Intraoperative blood loss (ml)	Wound complications (case)	Neurological complications (case)	Preoperative slippage rate (%)	Postoperative slippage rate (%)	Reduction rate (%)	Screw placement accuracy			

								Excellent (case)	Good (case)	Fair (case)	Poor (case)
Observation group	107.26±10.46	222.33±46.87	1	1	28.13±10.11	2.40±1.55	90.16±7.62	9	4	2	0
Control group	122.20±21.67	459.87±80.41	2	2	32.07±7.99	2.93±1.62	90.62±5.48	9	4	1	1
Statistical value	t=-2.403	t=-9.884			t=-1.183	t=-0.920	t=-0.189	X^2^=1.333			
P value	< 0.05	< 0.01	0.50^a^	0.50^a^	> 0.05	> 0.05	> 0.05	> 0.05			

***Note:***

a is Fisher exact probability method.

During the follow-up visit: the difference in the VAS score between the two groups had statistical significance 6 months after the operation (*P* < 0.01). The difference in the ODI between the two groups had no statistical significance at different time points (*P* > 0.05). VAS and ODI scores were improved at each time point compared with those before surgery. Table-III.

**Table-III:** VAS and ODI Scores of Patients in the Two Groups.

	Preoperative	1 week after the operation	1 month after the operation	3 months after the operation	6 months after the operation	1 year after the operation	F value	P value
VAS								
Observation group	6.73±1.33	4.26±1.22^a^	3.13±1.25^a^	2.00±1.20^a^	1.40±0.99^a^	0.93±0.70^a^	86.788	< 0.01
Control group	6.66±1.44	3.93±1.10^a^	3.20±0.77^a^	2.73±0.96^a^	2.40±0.63^a^	2.13±0.83^a^	46.486	< 0.01
t value	0.131	1.481	-0.176	-1.852	-3.307	-4.260		
P value	> 0.05	> 0.05	> 0.05	> 0.05	< 0.01	< 0.01		
ODI								
Observation group	58.67±8.16	29.47±4.98^a^	23.47±3.42^a^	18.80±2.93^a^	16.27±2.71^a^	8.80±3.69^a^	240. 063	< 0.01
Control group	59.20±7.24	31.20±4.89^a^	24.93±3.53^a^	21.47±3.74^a^	18.27±3.01^a^	10.53±3.74^a^	306.420	< 0.01
t value	-0.189	-0.962	-1.155	-2.173	-1.192	1.278		
P value	> 0.05	> 0.05	> 0.05	> 0.05	> 0.05	> 0.05		

***Note:*** VAS is visual analogue scale; ODI is Oswestry disability index;

a means that the difference from the preoperative values has statistical significance (P < 0.05).

## DISCUSSION

Lumbar spondylolisthesis is usually caused by degeneration or spondylolysis of the lumbar vertebra, often resulting in back pain, intermittent claudication and other symptoms. Operative treatment should be considered for patients in whom conservative treatment is ineffective and who have neurological impairment. Common operation methods include PLIF and TLIF.

PLIF is the most traditional lumbar decompression and fusion and has such advantages as wide decompression range and provision of three column spinal stability.^10^ TLIF was first proposed by Harms et al.^9^ It has been increasingly preferred by clinicians due to its advantages over the traditional PLIF, such as easier decompression, less nerve root pull and stimulation during the operation, lower probability of dural sac injury.^11^,^12^ However, because of the traditional TLIF has greater traction and stimulation on paraspinal muscles, it may cause residual pain in the back and soft tissues.^13^ With the development of technology, the application of percutaneous screw system has made MIS-TLIF more and more popular. Compared with conventional open TLIF, MIS-TLIF has such advantages as less amount of bleeding during the operation, shorter duration of operation, shorter length of stay (LOS) and fewer soft tissue injuries.^14^-^17^

During the follow-up visits in this study, there was no statistical difference in the ODI score between the two groups, while the VAS (lower back) score in the observation group was higher than that in the control group six months after the operation. These data indicate that MIS-TLIF under CT guidance has the same effect on the long-term neurological recovery of patients compared with open TLIF, while the postoperative lower back pain and discomfort due to the reduction of intraoperative lower back soft tissue injury is lower than open TLIF. The reduction of lower back pain is also more conducive to patients to carry out active functional exercise and accelerate the postoperative rehabilitation process. At the same time, we can see that there was no difference in neurological complications between the two groups, suggesting that MIS-TLIF under CT guidance did not increase the risk of neurological injury.

Most of the MIS-TLIF were operated by working channel and fluoroscopy assisted by C-arm. However, in practical use, the C-arm has certain limitations, thus the images of patients with spinal deformity are inadequate to provide guidance for surgical procedures.^18^ At the same time, because of the large number of C-arm operations, it needs to enter the operation area many times, which increases the risk of iatrogenic radiation exposure of surgeons.^19^ Compared with screwing by hand and X-ray perspective, the use of CT navigation system can improve the accuracy of screw placement in spinal surgery and lower the risk of damages to peripheral vital nerve vessels during screw placement.^20^,^21^ In a multicenter, prospective study, 353 patients were implanted with 1922 screws. About 2.5% of the screws were poorly placed, while only 1.8% needed to be repositioned.^22^ Zausinger et al.^23^ scholars also showed that CT navigation increased the accuracy of pedicle screw implantation, reduced the risk of surgery and improved the safety of surgery. Meanwhile, surgeons’ risk of radiation exposure is lowered.^24^ There is no difference in the reduction rate between the two groups, indicating that the CT-guided MIS-TLIF and the open TLIF have the same effect during the reduction of the vertebral body.

## CONCLUSIONS

In this study, the authors believe that, after continuous development, minimally invasive techniques have become increasingly mature. For single-segment lumbar spondylolisthesis, CT-guided MIS-TLIF surgery reduces operative time and intraoperative blood loss compared with open TLIF surgery. At the same time, due to less muscle and soft tissue destruction, MIS-TLIF is more advantageous in postoperative lower back pain. Furthermore, MIS-TLIF surgery did not increase the risk of perioperative complications. Nevertheless, this study also had such deficiencies as small sample size and short follow-up visits, which affected the evidence level of the study results. Statistical analysis of long-term follow-up of large samples is expected in the future.

### Authors’ Contributions

**RS,** **CW:** Designed this study and prepared this manuscript.

**JZ:** Collected and analyzed clinical data

**ZJ:** Revised and corrected this manuscript.

**CW:** Responsible for integrity and accuracy of the research.
